# Supplementary figures and images for: How many single-copy orthologous genes from whole genomes reveal deep gastropod relationships?
Source: PeerJ. 2022 Apr 18;10:e13285. doi: 10.7717/peerj.13285 (PMC9048639; doi:10.7717/peerj.13285)

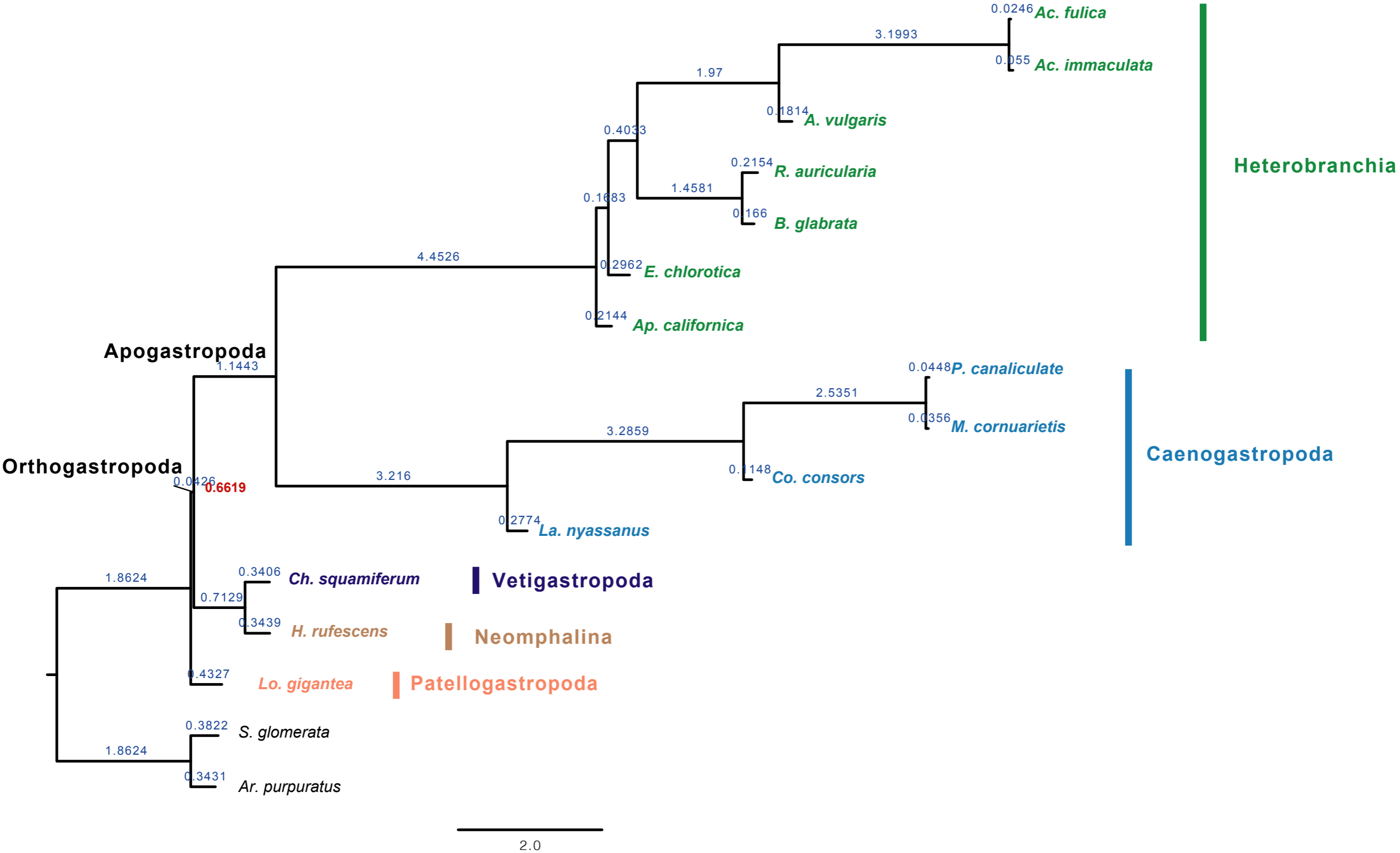

Supplement: Supplemental Information 1 — posterior probability (PP) is highlighted in red. Branch length is marked with blue numbers. [file peerj-10-13285-s001.pdf]

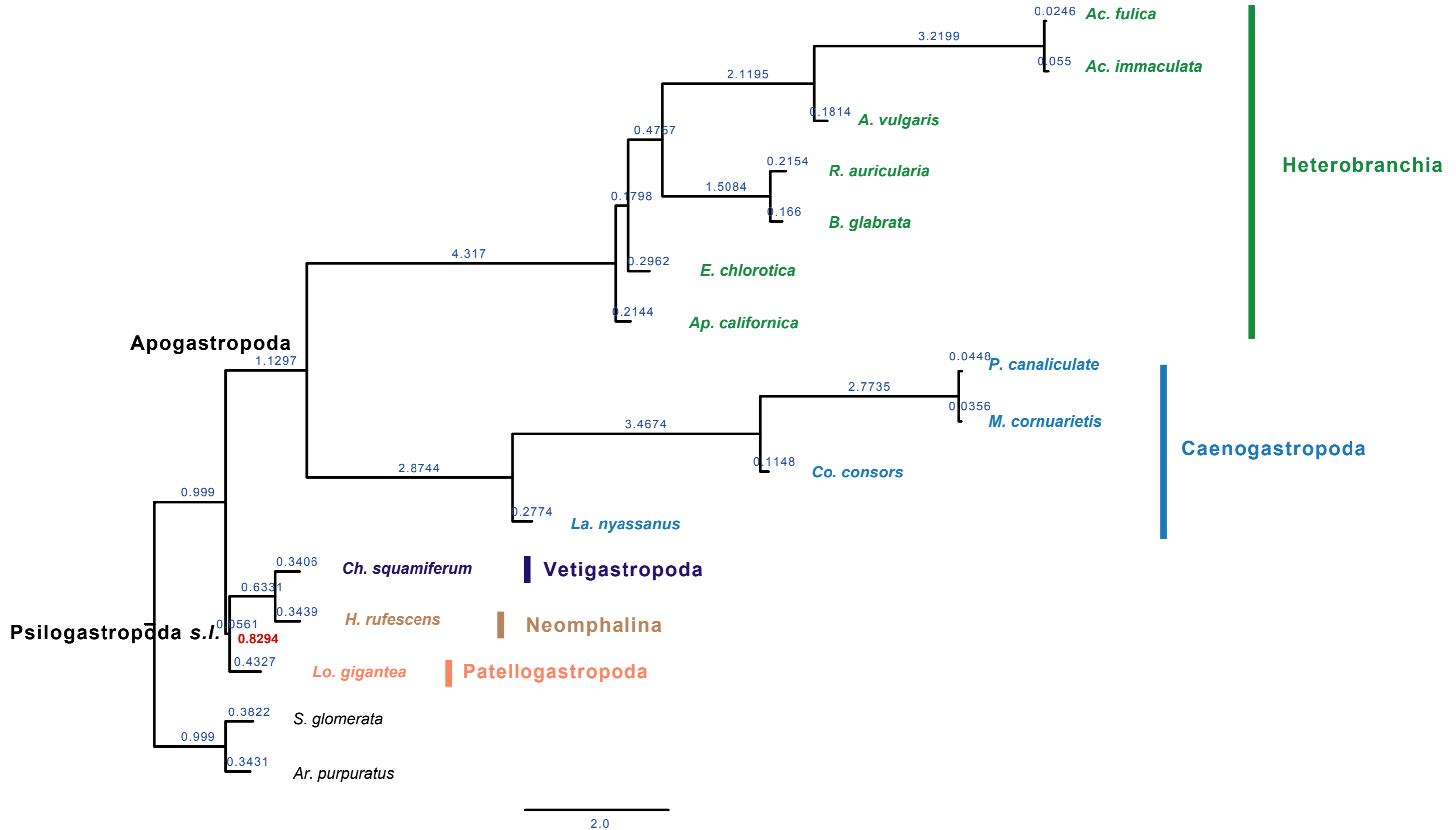

Supplement: Supplemental Information 2 — posterior probability (PP) is highlighted in red. Branch length is marked with blue numbers. [file peerj-10-13285-s002.pdf]

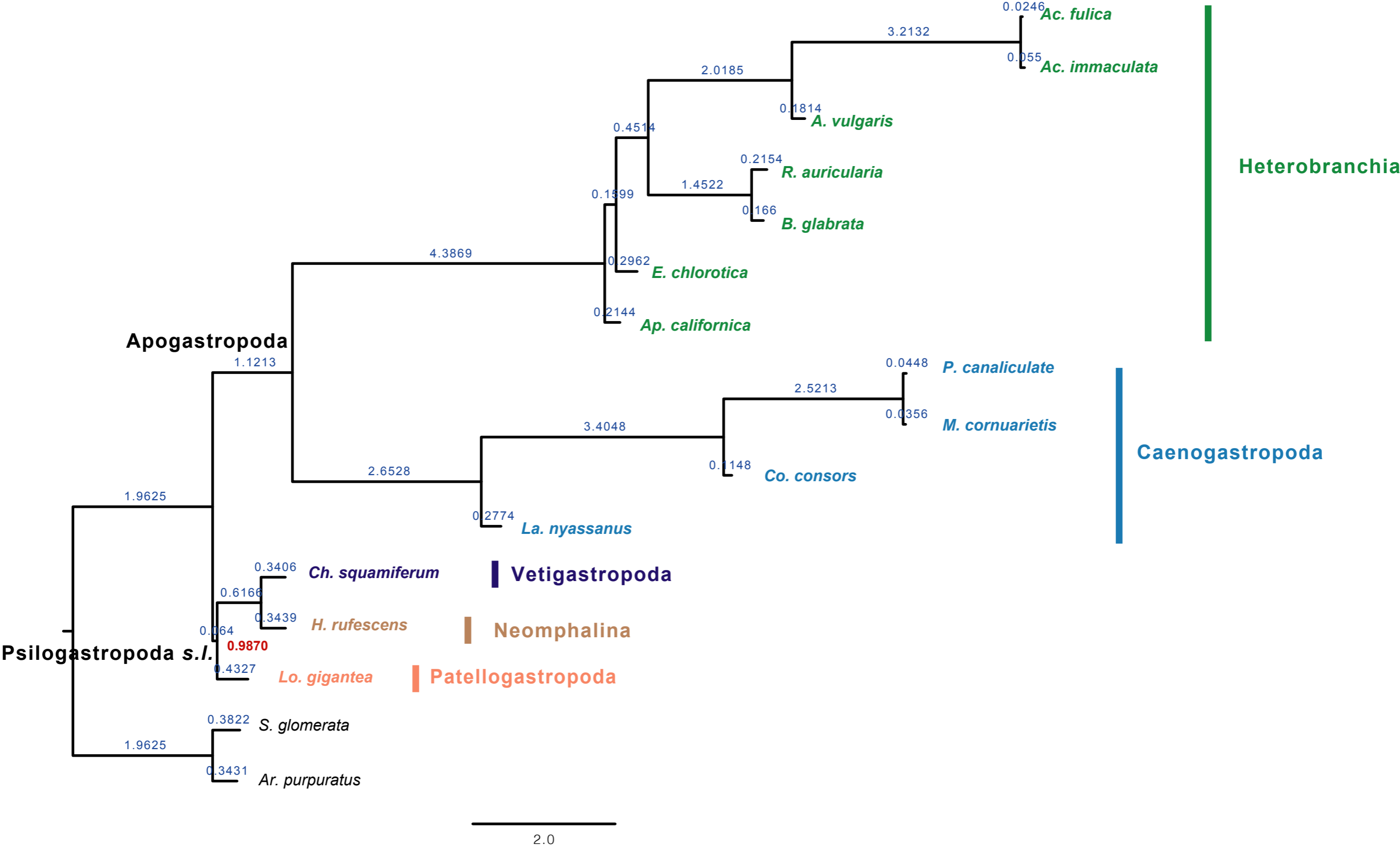

Supplement: Supplemental Information 3 — posterior probability (PP) is highlighted in red. Branch length is marked with blue numbers. [file peerj-10-13285-s003.pdf]

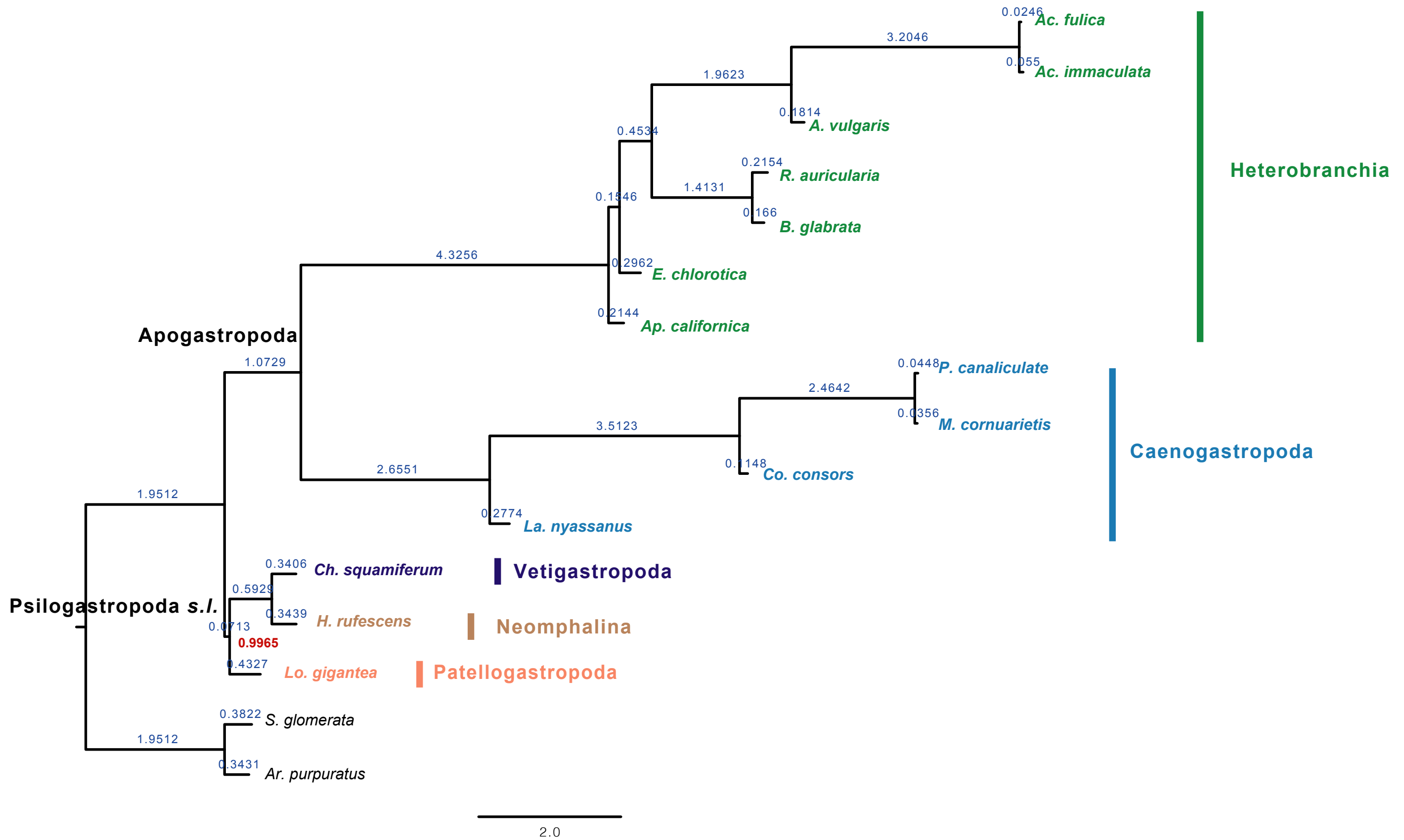

Supplement: Supplemental Information 4 — posterior probability (PP) is highlighted in red. Branch length is marked with blue numbers. [file peerj-10-13285-s004.pdf]
