# Supplementary material for: How many single-copy orthologous genes from whole genomes reveal deep gastropod relationships?
Source: PeerJ. 2022 Apr 18;10:e13285. doi: 10.7717/peerj.13285 (PMC9048639; doi:10.7717/peerj.13285)
Supplement: Supplemental Information 5 — The best-fit model according to BIC: JTT+F+R5. AIC, w-AIC: Akaike information criterion scores and weights. AICc, w-AICc: Corrected AIC scores and weights. BIC, w-BIC: Bayesian information criterion scores and weights. Plus signs denote the 95% confidence sets. Minus signs denote significant exclusion. [file peerj-10-13285-s005.docx]

**Table S1. List of models sorted by BIC scores.** The best-fit model according to BIC: JTT+F+R5. AIC, w-AIC: Akaike information criterion scores and weights. AICc, w-AICc: Corrected AIC scores and weights. BIC, w-BIC: Bayesian information criterion scores and weights. Plus signs denote the 95% confidence sets. Minus signs denote significant exclusion.

| Model | LogL | AIC | w-AIC | AICc | w-AICc | BIC | w-BIC |
| --- | --- | --- | --- | --- | --- | --- | --- |
| JTT+F+R5 | -9617668.9 | 19235445.82 | +0.12 | 19235445.8 | +0.12 | 19236069.55 | +1 |
| JTT+F+R6 | -9617664.9 | 19235441.83 | +0.88 | 19235441.8 | +0.88 | 19236088.66 | -7E-05 |
| LG+F+R6 | -9617709.1 | 19235530.26 | -5.52E-20 | 19235530.3 | -5.52E-20 | 19236177.09 | -4E-24 |
| LG+F+R5 | -9617725.7 | 19235559.35 | -2.67E-26 | 19235559.4 | -2.67E-26 | 19236183.08 | -2E-25 |
| JTTDCMut+F+R5 | -9617961.7 | 19236031.35 | -8.58E-129 | 19236031.4 | -8.59E-129 | 19236655.07 | -7E-128 |
| JTTDCMut+F+R6 | -9617957.8 | 19236027.5 | -5.86E-128 | 19236027.5 | -5.86E-128 | 19236674.33 | -4E-132 |
| WAG+F+R5 | -9632763.6 | 19265635.28 | -0 | 19265635.3 | -0 | 19266259.01 | -0 |
| WAG+F+R6 | -9632758.7 | 19265629.37 | -0 | 19265629.4 | -0 | 19266276.2 | -0 |
| VT+F+R5 | -9634284.1 | 19268676.19 | -0 | 19268676.2 | -0 | 19269299.92 | -0 |
| VT+F+R6 | -9634282.7 | 19268677.47 | -0 | 19268677.5 | -0 | 19269324.3 | -0 |
| JTT+R5 | -9639564.2 | 19279198.49 | -0 | 19279198.5 | -0 | 19279602.75 | -0 |
| JTT+R6 | -9639560.2 | 19279194.34 | -0 | 19279194.3 | -0 | 19279621.71 | -0 |
| JTTDCMut+R5 | -9639889.1 | 19279848.28 | -0 | 19279848.3 | -0 | 19280252.55 | -0 |
| JTTDCMut+R6 | -9639885.5 | 19279845.01 | -0 | 19279845 | -0 | 19280272.38 | -0 |
| rtREV+F+R5 | -9644512.4 | 19289132.85 | -0 | 19289132.9 | -0 | 19289756.57 | -0 |
| rtREV+F+R6 | -9644502.4 | 19289116.77 | -0 | 19289116.8 | -0 | 19289763.6 | -0 |
| LG+R5 | -9646067.8 | 19292205.66 | -0 | 19292205.7 | -0 | 19292609.93 | -0 |
| LG+R6 | -9646056.6 | 19292187.19 | -0 | 19292187.2 | -0 | 19292614.56 | -0 |
| LG+R4 | -9646466.7 | 19292999.48 | -0 | 19292999.5 | -0 | 19293380.65 | -0 |
| VT+R5 | -9648879.4 | 19297828.73 | -0 | 19297828.7 | -0 | 19298233 | -0 |
| VT+R6 | -9648878.1 | 19297830.14 | -0 | 19297830.1 | -0 | 19298257.51 | -0 |
| LG+I+G4 | -9649218.2 | 19298494.43 | -0 | 19298494.4 | -0 | 19298829.4 | -0 |
| LG+G4 | -9651046.4 | 19302148.89 | -0 | 19302148.9 | -0 | 19302472.31 | -0 |
| LG+R3 | -9651370 | 19302801.95 | -0 | 19302802 | -0 | 19303160.02 | -0 |
| WAG+R5 | -9653905.2 | 19307880.38 | -0 | 19307880.4 | -0 | 19308284.65 | -0 |
| WAG+R6 | -9653900.2 | 19307874.35 | -0 | 19307874.4 | -0 | 19308301.72 | -0 |
| Dayhoff+F+R6 | -9662727 | 19325565.98 | -0 | 19325566 | -0 | 19326212.8 | -0 |
| Dayhoff+F+R5 | -9662744.5 | 19325596.92 | -0 | 19325596.9 | -0 | 19326220.64 | -0 |
| DCMut+F+R6 | -9662790.8 | 19325693.56 | -0 | 19325693.6 | -0 | 19326340.39 | -0 |
| DCMut+F+R5 | -9662808.2 | 19325724.43 | -0 | 19325724.4 | -0 | 19326348.16 | -0 |
| mtInv+F+R6 | -9671163.6 | 19342439.25 | -0 | 19342439.3 | -0 | 19343086.08 | -0 |
| mtInv+F+R5 | -9671195 | 19342498.02 | -0 | 19342498 | -0 | 19343121.75 | -0 |
| cpREV+F+R6 | -9684203.1 | 19368518.1 | -0 | 19368518.1 | -0 | 19369164.93 | -0 |
| cpREV+F+R5 | -9684222.5 | 19368553.09 | -0 | 19368553.1 | -0 | 19369176.81 | -0 |
| PMB+F+R5 | -9693167.3 | 19386442.61 | -0 | 19386442.6 | -0 | 19387066.34 | -0 |
| PMB+F+R6 | -9693165 | 19386441.97 | -0 | 19386442 | -0 | 19387088.8 | -0 |
| LG+R2 | -9696350.1 | 19392758.18 | -0 | 19392758.2 | -0 | 19393093.15 | -0 |
| Blosum62+F+R5 | -9697606.1 | 19395320.19 | -0 | 19395320.2 | -0 | 19395943.92 | -0 |
| Blosum62+F+R6 | -9697604.4 | 19395320.71 | -0 | 19395320.7 | -0 | 19395967.54 | -0 |
| mtMet+F+R6 | -9710893.7 | 19421899.45 | -0 | 19421899.5 | -0 | 19422546.28 | -0 |
| mtMet+F+R5 | -9710951.4 | 19422010.89 | -0 | 19422010.9 | -0 | 19422634.61 | -0 |
| Dayhoff+R6 | -9711459.9 | 19422993.79 | -0 | 19422993.8 | -0 | 19423421.16 | -0 |
| Dayhoff+R5 | -9711476.2 | 19423022.32 | -0 | 19423022.3 | -0 | 19423426.58 | -0 |
| DCMut+R6 | -9711641.1 | 19423356.17 | -0 | 19423356.2 | -0 | 19423783.54 | -0 |
| DCMut+R5 | -9711656.9 | 19423383.85 | -0 | 19423383.9 | -0 | 19423788.12 | -0 |
| mtZOA+F+R6 | -9714727.9 | 19429567.84 | -0 | 19429567.9 | -0 | 19430214.67 | -0 |
| mtZOA+F+R5 | -9714990.9 | 19430089.89 | -0 | 19430089.9 | -0 | 19430713.62 | -0 |
| rtREV+R5 | -9722048.7 | 19444167.32 | -0 | 19444167.3 | -0 | 19444571.59 | -0 |
| rtREV+R6 | -9722039.1 | 19444152.24 | -0 | 19444152.2 | -0 | 19444579.61 | -0 |
| PMB+R5 | -9725572.9 | 19451215.83 | -0 | 19451215.8 | -0 | 19451620.09 | -0 |
| PMB+R6 | -9725569.1 | 19451212.16 | -0 | 19451212.2 | -0 | 19451639.53 | -0 |
| cpREV+R5 | -9728657.4 | 19457384.89 | -0 | 19457384.9 | -0 | 19457789.16 | -0 |
| cpREV+R6 | -9728646.3 | 19457366.68 | -0 | 19457366.7 | -0 | 19457794.05 | -0 |
| mtREV+F+R6 | -9732086.7 | 19464285.49 | -0 | 19464285.5 | -0 | 19464932.32 | -0 |
| mtREV+F+R5 | -9732158.3 | 19464424.53 | -0 | 19464424.5 | -0 | 19465048.25 | -0 |
| Blosum62+R5 | -9733212.2 | 19466494.45 | -0 | 19466494.4 | -0 | 19466898.71 | -0 |
| Blosum62+R6 | -9733209.4 | 19466492.76 | -0 | 19466492.8 | -0 | 19466920.13 | -0 |
| FLU+F+R6 | -9734824.9 | 19469761.81 | -0 | 19469761.8 | -0 | 19470408.64 | -0 |
| FLU+F+R5 | -9735056.2 | 19470220.38 | -0 | 19470220.4 | -0 | 19470844.1 | -0 |
| HIVb+F+R6 | -9753619.1 | 19507350.27 | -0 | 19507350.3 | -0 | 19507997.1 | -0 |
| HIVb+F+R5 | -9753668.6 | 19507445.14 | -0 | 19507445.1 | -0 | 19508068.87 | -0 |
| FLU+R6 | -9787731.1 | 19575536.17 | -0 | 19575536.2 | -0 | 19575963.54 | -0 |
| FLU+R5 | -9787972.7 | 19576015.44 | -0 | 19576015.4 | -0 | 19576419.71 | -0 |
| mtART+F+R6 | -9791886.5 | 19583885.01 | -0 | 19583885 | -0 | 19584531.84 | -0 |
| mtART+F+R5 | -9792490.8 | 19585089.63 | -0 | 19585089.6 | -0 | 19585713.36 | -0 |
| mtVer+F+R6 | -9812605.2 | 19625322.36 | -0 | 19625322.4 | -0 | 19625969.19 | -0 |
| mtVer+F+R5 | -9812686.5 | 19625480.94 | -0 | 19625481 | -0 | 19626104.67 | -0 |
| HIVb+R6 | -9836157.5 | 19672389.08 | -0 | 19672389.1 | -0 | 19672816.44 | -0 |
| HIVb+R5 | -9836193 | 19672456.01 | -0 | 19672456 | -0 | 19672860.27 | -0 |
| LG+I | -9853377.3 | 19706810.56 | -0 | 19706810.6 | -0 | 19707133.97 | -0 |
| mtMAM+F+R6 | -9874496.7 | 19749105.4 | -0 | 19749105.4 | -0 | 19749752.23 | -0 |
| mtMAM+F+R5 | -9875083.9 | 19750275.82 | -0 | 19750275.8 | -0 | 19750899.54 | -0 |
| HIVw+F+R6 | -9955266.9 | 19910645.83 | -0 | 19910645.8 | -0 | 19911292.66 | -0 |
| HIVw+F+R5 | -9955368.8 | 19910845.67 | -0 | 19910845.7 | -0 | 19911469.4 | -0 |
| mtZOA+R6 | -10028697 | 20057467.72 | -0 | 20057467.7 | -0 | 20057895.09 | -0 |
| mtZOA+R5 | -10028812 | 20057693.08 | -0 | 20057693.1 | -0 | 20058097.35 | -0 |
| mtMet+R6 | -10106713 | 20213500.58 | -0 | 20213500.6 | -0 | 20213927.95 | -0 |
| mtMet+R5 | -10106771 | 20213612.8 | -0 | 20213612.8 | -0 | 20214017.07 | -0 |
| LG | -10111112 | 20222278.9 | -0 | 20222278.9 | -0 | 20222590.76 | -0 |
| mtREV+R6 | -10125829 | 20251731.26 | -0 | 20251731.3 | -0 | 20252158.63 | -0 |
| mtREV+R5 | -10125882 | 20251834.98 | -0 | 20251835 | -0 | 20252239.25 | -0 |
| HIVw+R6 | -10143980 | 20288033.12 | -0 | 20288033.1 | -0 | 20288460.49 | -0 |
| HIVw+R5 | -10144105 | 20288279.62 | -0 | 20288279.6 | -0 | 20288683.89 | -0 |
| mtInv+R6 | -10171857 | 20343787.74 | -0 | 20343787.7 | -0 | 20344215.11 | -0 |
| mtInv+R5 | -10171879 | 20343827.44 | -0 | 20343827.4 | -0 | 20344231.71 | -0 |
| mtART+R6 | -10186387 | 20372848.9 | -0 | 20372848.9 | -0 | 20373276.27 | -0 |
| mtART+R5 | -10187267 | 20374603.08 | -0 | 20374603.1 | -0 | 20375007.35 | -0 |
| mtVer+R6 | -10194988 | 20390050.56 | -0 | 20390050.6 | -0 | 20390477.93 | -0 |
| mtVer+R5 | -10195102 | 20390273.7 | -0 | 20390273.7 | -0 | 20390677.97 | -0 |
| mtMAM+R6 | -10283473 | 20567019.46 | -0 | 20567019.5 | -0 | 20567446.83 | -0 |
| mtMAM+R5 | -10283866 | 20567801.1 | -0 | 20567801.1 | -0 | 20568205.37 | -0 |
